# Corrigendum to “DNA methylation markers for oral pre-cancer progression: A critical review” [Oral Oncology 53 (2015) 1–9]

**DOI:** 10.1016/j.oraloncology.2016.07.011

**Published:** 2016-09

**Authors:** Krithiga Shridhar, Gagandeep Kaur Walia, Aastha Aggarwal, Smriti Gulati, A.V. Geetha, Dorairaj Prabhakaran, Preet K. Dhillon, Preetha Rajaraman

**Affiliations:** a Centre for Chronic Conditions and Injuries, Public Health Foundation of India, Gurgaon, Haryana, India; b Centre for Chronic Disease Control, Gurgaon, Haryana, India; c London School of Hygiene and Tropical Medicine, London, United Kingdom; d Center for Global Health, National Cancer Institute, NIH, DHHS, Bethesda, Maryland, USA

The authors regret the error published in Introduction Section on Page 1, Line 7: “Over a million new cases are reported every year from more developed regions of the World [1], more so among young adults [4,5]”. Please note the corrected statement with respect to the above mentioned line: “Over a hundred thousand new cases are reported every year from more developed regions of the World [1], more so among young adults [4,5]”.

The authors would like to apologise for any inconvenience caused.

